# Evidence for deficits in behavioural and physiological responses in aged mice relevant to the psychiatric symptom of apathy

**DOI:** 10.1177/23982128211015110

**Published:** 2021-05-25

**Authors:** Megan G. Jackson, Stafford L. Lightman, Gary Gilmour, Hugh Marston, Emma S. J. Robinson

**Affiliations:** 1 School of Physiology, Pharmacology and Neuroscience, University of Bristol, Bristol, UK; 2 Bristol Medical School: Translational Health Sciences, University of Bristol, Bristol, UK; 3 Eli Lilly & Co. Ltd, Surrey, UK

**Keywords:** Ageing, apathy, emotional blunting, motivation, stress reactivity

## Abstract

Apathy is widely reported in patients with neurological disorders or post viral infection but is also seen in otherwise-healthy aged individuals. This study investigated whether aged male mice express behavioural and physiological changes relevant to an apathy phenotype. Using measures of motivation to work for reward, we found deficits in the progressive ratio task related to rate of responding. In an effort-related decision-making task, aged mice were less willing to exert effort for high value reward. Aged mice exhibited reduced reward sensitivity but also lower measures of anxiety in the novelty supressed feeding test and an attenuated response to restraint stress with lower corticosterone and reduced paraventricular nucleus c-fos activation. This profile of affective changes did not align with those observed in models of depression but suggested emotional blunting. In a test of cognition (novel object recognition), aged mice showed no impairments, but activity was lower in a measure of exploration in a novel environment. Together, these data suggest aged mice show changes across the domains of motivated behaviour, reward sensitivity and emotional reactivity and may be a suitable model for the pre-clinical study of the psychiatric symptom of apathy.

## Introduction

Ageing is associated with widespread physiological changes, including disruptions to normal behaviour. A prevalent behavioural change is the onset of the psychiatric symptom, apathy (Brodaty et al., 2009), which is defined as a quantitative reduction in self-generated or voluntary behaviours (Levy and Czernecki, 2006). While apathy is a common feature of neurodegenerative diseases, and in some people following viral infections (Kamat et al., 2012), it is also seen in otherwise-healthy ageing. Apathy can have a profound effect on the daily functioning of the individual and the caregiver. It is associated with cognitive decline, nutritional deficit and an overall poorer quality of life as well as significant caregiver stress (Gerritsen et al., 2005; Ishii et al., 2009). It therefore represents an important potential target for pharmacological interventions.

While generally considered a motivational disorder, the work of Marin, Levy and Dubois has conceptualised apathy into three domains: an emotional/affective component, an auto-activation/behavioural component and a cognitive component (Levy and Dubois, 2006). Efforts have been made to map these components onto distinct brain circuits, particularly those of the frontal cortex-basal ganglia (Levy and Dubois, 2006), though there have been few studies to understand apathy in the context of normal ageing or using animal models. The development of an approach to study these domains of apathy in non-human species could facilitate insight into its underlying neurobiology and potentially elucidate therapeutic targets. Clinical assessment of apathy is traditionally conducted using self-report or questionnaire-based methods (e.g. Apathy Evaluation Scale (Marin et al., 1991) and Lille Apathy Rating Scale (Sockeel et al., 2006)). These forms of assessment rely on the individual to recognise changes in their own behaviour and have limited sensitivity to track changes in behaviour over time. These subjective methods cannot be directly translated to rodent tasks. When considering animal models in psychiatry, particularly where a diagnosis is related to deficits in several symptom domains, it is useful to consider the framework set out in RDoC (Research Domains of Criteria, (Cuthbert, 2015)). Rather than looking at the effects of ageing on one specific area in isolation, using a battery of measures can provide a more detailed insight into the changes which are present across multiple domains. Where behavioural tasks involve more than one underlying construct, using a test battery will also benefit the overall interpretation of the findings.

In this study, we tested a cohort of normal, healthy aged mice using behavioural assays specifically designed to quantify domains of emotional behaviour, motivation, reward and cognition. In contrast to previous studies, we tested the same animals across different tasks so we could achieve an overall profile of deficits and then relate this back to what has been observed in human ageing and apathy. We selected tasks based on RDoC and where, as much as possible, translational analogues were available.

To investigate changes in motivation, we chose two different behavioural tasks which have been successfully back translated to human studies: the progressive ratio (PR) task (Chelonis et al., 2011; Richardson and Roberts, 1996) and effort for reward task (EfR) (Heath et al., 2015; Salamone et al., 1991; Treadway et al., 2009). PR requires the rodent to expend increasing amounts of effort for each subsequent reward with the point when the animal stops responding, the breakpoint, used as a measure of motivation. In the EfR, the animal is given a choice between a high effort, high value reward option or a low effort, low value reward option providing a measure of effort-related choice behaviour which is sensitive to changes in motivational state and manipulations of dopaminergic transmission (Salamone et al., 2018).

Emotional blunting, defined as a diminished response to emotionally salient stimuli, is a core feature of apathy but is often overlooked in favour of effort-based paradigms to study motivation, particularly in rodent models (Magnard et al., 2016). Some groups have reported changes in anxiety/depression behaviour in models of neurodegenerative disorders (Taylor et al., 2010) and more recently, healthy ageing (Shoji and Miyakawa, 2019), and changes in fear processing have been used as a model of emotional blunting in a rodent model of schizophrenia (Pietersen et al., 2007). One of the challenges is the limitations associated with animal models of anxiety and depression (Commons et al., 2017), with conventional measures such as behavioural despair used to predict antidepressant efficacy but with poor validity in terms of quantifying phenotypic changes (Reardon, 2019) and hence may have limited value in relation to emotional blunting. We chose to use the novelty supressed feeding test (NSFT) and then quantify both baseline and stress-induced corticosterone and post-mortem c-Fos expression to investigate the effects of ageing on stress reactivity at a behavioural, physiological and cellular level. The NSFT has been used to investigate stress-related behaviours in depression and anxiety research in phenotypic models, and studies in models such as early life adversity (ELA) has shown both behavioural changes in the NSFT and stress reactivity at a physiological and molecular level (Stuart et al., 2019; Uchida et al., 2010). We also tested animals using the sucrose preference test to quantify reward sensitivity and performed a novel object recognition test to look at their associative recognition memory. By assessing the aged mouse phenotype across these different domains and comparing the findings to those seen in other models, for example, depression, we predicted being able to show a distinct behavioural profile and establish a test battery which could be used to study the relevant underlying neurobiology of apathy.

## Methods

### Subjects

**Figure 1.**
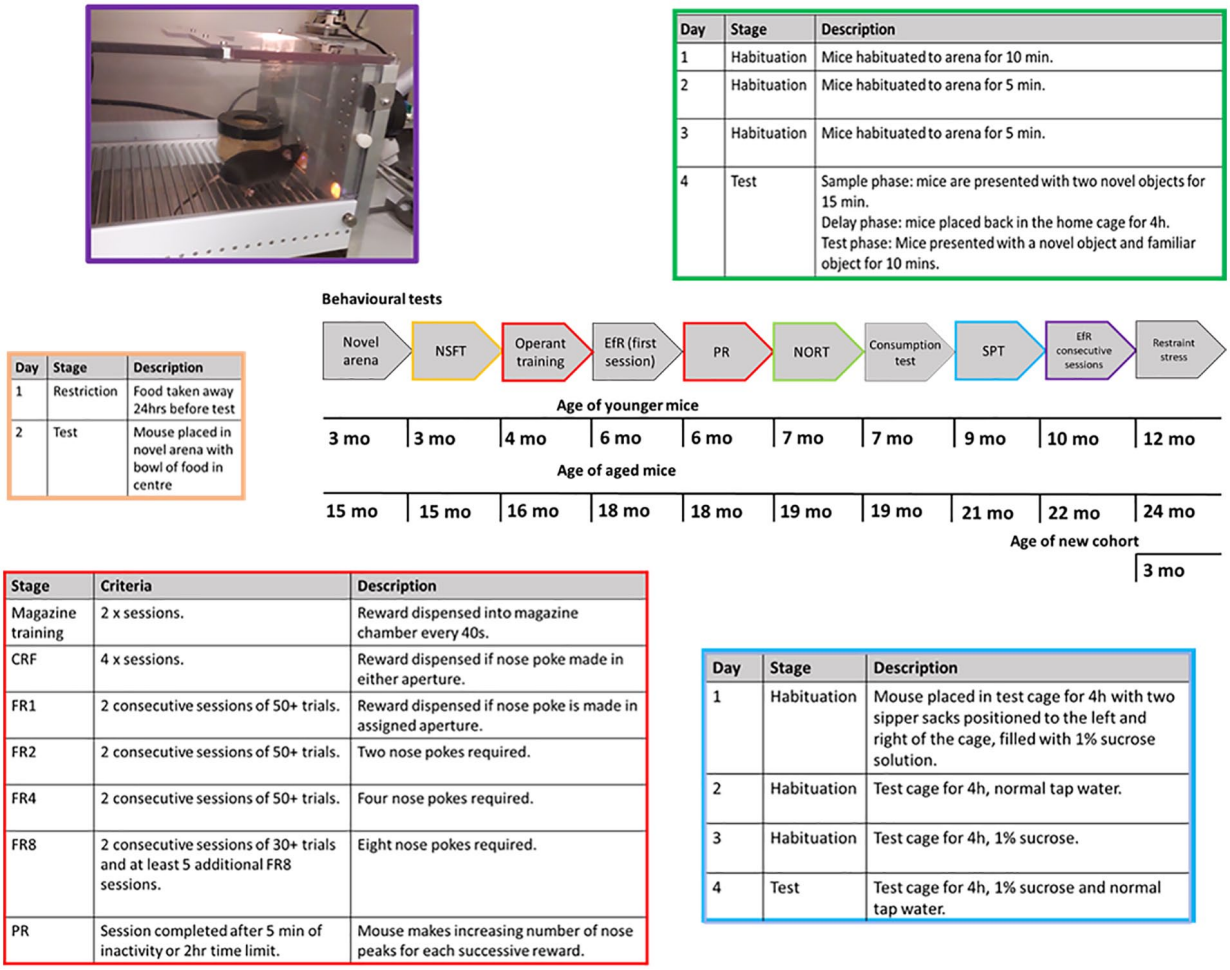
Experimental timeline and training protocols. Experimental timeline illustrating the different behavioural procedures used and when these occurred. Where more complex behavioural tasks have been used, additional information about training is also included and colour coded to link to the specific task within the timeline. CRF: continuous reinforcement, EfR: effort for reward, FR: fixed ratio, NORT: novel object recognition test, NSFT: novelty supressed feeding test, PR: progressive ratio, SPT: sucrose preference test.

A cohort of 12 aged male C57bl/6J mice (15 mo at experiment onset and 24 mo by end of experimentation, 31.6–38.9 g at onset and 33.6–40.0 g by end) supplied by Eli Lilly and a cohort of C57bl/6J strain and sex-matched controls (3 mo at experiment onset and 12 mo by end of experimentation, 21.8–27.6 g at experiment onset and 30.5–32.4 g by end) supplied by Charles River were used. Sample size was based on previous behavioural studies using both spontaneous behavioural assays such as the open-field arena and operant methods (Gourley et al., 2016; Rex et al., 1998). However, this type of ageing work is novel, and effect sizes may be smaller than those more typically seen with manipulations in these assays. Prior to arrival in Bristol, aged mice were group-housed in enriched caging and fed a restricted diet of 3 g to promote healthy ageing. On arrival in Bristol, we noticed significant in-fighting in the aged group and as a consequence, all mice were individually housed in open-top cages. Cages were enriched with a plastic house, cardboard tube and wooden chew block. They were kept in temperature-controlled conditions (21°C) and a 12:12-h light–dark cycle (lights OFF at 08:15, lights ON at 20:15). Standard laboratory chow (Purina, UK) was provided *ad libitum*, apart from during operant training where mice were fed a restricted diet of 2 g chow per mouse. Weights were monitored at least once a week and maintained to at least 85% of their free feeding weight relative to their normal growth curve. Water was provided *ad libitum*. The same cohorts of animals were used for all studies and a timeline indicating the order of testing is given in Figure 1. As the tests were performed sequentially, there is the potential that increasing age may have affected some measures, and hence the design randomised the tasks between measures of motivation-related and emotional behaviour. It should be noted that this design does not fully mitigate an effect of order of testing or increasing age of the animals. Within each behavioural assay, age was counter-balanced to account for time of day differences. Where possible, the experimenter was blind to age group, though this was not always possible due to obvious physical differences between age groups. Aged mice were checked daily for changes in health that would impair performance in behavioural tasks, including overt changes in motoric function. If this occurred, mice were removed from the study. However, no animals were excluded based on health. All experiments took place in the animals’ active phase and were performed in accordance with the Animals (Scientific Procedures) Act (United Kingdom) 1986 and were approved by the University of Bristol Animal Welfare and Ethical Review Body (AWERB).

### Exploration of a novel arena

Mice were placed in a novel, circular open-field arena, 85 cm in diameter under red lighting. Movement was captured for 15 min using a Logitech HD Pro Webcam c920 suspended 1 m above the arena. Videos were analysed using Ethovision Xt10 software (Noldus Information Technology, Wageningen) and total distance travelled (cm) and velocity (cm/s) were output.

### Novelty suppressed feeding test

The protocol used was similar to that reported by Shephard and Broadhurst (1982). Mice were food deprived for 24 h before being placed in the left-hand corner of a novel arena (clear Perspex, 40 cm × 40 cm and lined with sawdust). A ceramic bowl of food was placed in the centre of the arena. Time taken for the mice to approach the bowl and to eat was manually recorded. Faecal pellets were removed after each mouse, and the sawdust was shaken to redistribute odour cues but reduce the aversive nature of the context, the food bowl was cleaned, and fresh food added between mice.

### Operant training

Training was similar to that reported by Stuart et al. (2019). Mice were trained in sound-proof operant boxes (Med Associates Inc) which were run on Klimbic software (Conclusive Solutions Ltd., UK). Each operant box consisted of two nose poke apertures positioned either side of a centrally located food magazine. The magazine was connected to a reward pellet dispenser (20 mg rodent tablet, TestDiet, Sandown). Mice were run once per day during their active phase (9:00–17:00). Mice first learned to associate the magazine with the delivery of a reward pellet (one pellet every 40 s) over two 30-min sessions. Mice then progressed to continuous reinforcement training (CRF), where a response made in either the left or right assigned nose poke aperture resulted in a single reward pellet. The magazine was illuminated until the mouse collected the pellet. In the third stage of training, the mice were required to advance through ascending fixed ratios (FRs) of reinforcement, making responses in either the left or right aperture only (counter-balanced across cohorts). The mice progressed through FR1, 2, 4 and 8, where the number refers to the number of nose pokes into the active aperture required for the delivery of one reward pellet. Mice completed each FR level when they obtained 50+ pellets over two consecutive sessions until FR8 where criteria was 30+ pellets over two consecutive sessions. Once all mice were trained, mice then completed a minimum of five additional FR8 sessions to manage differences in time to train (Figure 1).

### EfR task

Directly after FR training was completed, the mice underwent a single FR8 session with access to low value, *ad libitum* powdered standard laboratory chow presented in a pot placed in front of the inactive nose poke aperture similar to that previously described by (Salamone et al., 1991). Animals were required to make a sequence of eight nose pokes to obtain a single reward pellet (20 mg rodent tablet, TestDiet, Sandown) which represents the high effort, high reward option. The freely available chow represents the low effort, low value reward option as it is their standard diet and lacks the higher sucrose content of the reward pellets. The pot was accessed via a 0.5 inch hole in the lid. Chow consumed was measured using change in weight of chow pre and post session, in gramme. Later in the behavioural battery (Figure 1), mice were re-tested in the EfR task, but this time, over five consecutive sessions.

### PR task

Mice were tested in a PR task, in which each successive reward (*n*) required an increasing number of nose pokes using the algorithm F(*n*) = 5 × EXP(0.2*n*)−5 (Roberts and Richardson, 1992). A PR session consisted of a maximum of 100 trials or 120 min and included a 1-s intertrial interval. 5 mins of inactivity ended the session. This test was conducted under both *ad libitum* and food-restricted conditions. Breakpoint was defined as the last ratio competed before 5 min period of inactivity. Mice underwent one session of the PR task under each feeding condition. A PR session was preceded by an FR8 session to check for stability in performance. Preceding the described PR conditions, mice were tested with a PR session that lasted a maximum of 60 mins or 10 mins of inactivity. However, it was clear a breakpoint would not be reached under these conditions (data not shown).

### Consumption test

Mice were food restricted overnight, and the following day were presented with free access to either powdered chow or reward pellets for 10 min over two different days in the home cage. Total amount consumed in gramme was calculated.

### Novel object recognition test

This protocol was similar to that previously developed by Ennaceur and Delacour (1988). Mice were habituated to a Perspex arena (40 cm ×40 cm) lined with paper liner for 10 min (Day 1) or 5 min (Days 2 and 3). This arena was differentiated from the NSFT arena by changing the room in which experiment was run and changing the sawdust floor to a paper floor to change context. On Day 4, animals were tested using a sample phase where each mouse was presented with two novel objects for 15 mins. Mice were returned to their home cage during the 4-h delay phase before being returned to the same arena for the test phase. Each animal was presented with both a novel and familiar object for 10 mins (Figure 1). During both the sample and test phase, exploration (defined here as the animal pointing its nose towards the object at a distance of approximately <2 cm) was captured using a Logitech c920 webcam and then scored manually using DOSBox 0.74 software. Criteria for inclusion in the analysis was 20+ s of total exploration in the sample phase as described by Lueptow (2017). A discrimination ratio was calculated using (time spent exploring novel object – time spent exploring familiar object)/(time spent exploring novel object + time spent exploring familiar object).

### Sucrose preference test

The protocol used was similar to that by Willner et al. (1987). Mice were water restricted overnight for ~16 h before both the habituation and test sessions. On Days 1 and 3, the mice were placed into test cages which contained sawdust and a cardboard tube and were presented with a 1% sucrose solution in two sipper sacks (Edstrom-Avidity Science) with drip-free drinking valves, placed to the left and the right of the cage. On Day 2, the mice were presented with two sipper sacks containing normal tap water. On the test day, they were presented with one sipper sack containing 1% sucrose solution and the other containing tap water (Figure 1). Position was counter-balanced across the cohort and swapped at 1 h and 2 h. The habituation and test sessions lasted 4 h, and liquid consumed was weighed at the 1-, 2- and 4-h time points. Sucrose preference was calculated using (total amount of sucrose consumed/(total sucrose + total water consumed)) × 100.

### Acute restraint stress

At the end of behavioural experiments, groups were approximately 12 months and 24 months old. As such, an additional cohort was brought in as a young group. *N* = 12 male C57bl/6J mice from Charles River (3 mo at experiment onset) were singly housed in open-top cages upon arrival at the unit. Mice were kept in the same lighting conditions as described above and were given the same cage enrichment. Food and water were provided *ad libitum.* Mice spent 1 month in the unit before this experiment and were handled for 3 weeks to prevent potential confounds of stress from handling. The new mice weighed 23.2–26.9 g at the experiment end. One mouse from the oldest group died before this final experiment.

Mice were individually restrained in a restraint tube (Ad Instruments Ltd), and their tail warmed for 3 minutes on a heat pad to promote blood flow in the tail vein. The tail vein was then opened with a 25G needle (Sigma-Aldrich, Germany) and blood was collected using a Mitra Microsampling sponge (Neoteryx, USA), with a calculated average blood wicking volume of 10 µL. The mouse was left in the restraint tube for a further 24 mins, before having the tail vein warmed again for 3 mins and another blood sample was taken. Throughout the period of restraint and blood sampling, the animals were not in olfactory or auditory contact with other mice and the restraint tube was cleaned fully between animals. Sampling was also counter-balanced across the different groups. As an additional measure faecal pellets during testing were counted as it has previously been shown psychological stress significantly increases faecal output in rodents (Mönnikes et al., 1993). Sampling took place between zeitgeber time (ZT) 17–20. Blood samples were stored at room temperature with a bag of desiccant until analysis.

### Mass spectrometry analysis of corticosterone

Following sample extraction corticosterone levels were analysed using high-performance liquid chromatography/electrospray ionisation tandem mass spectrometry (HPLC-ES/MS-MS) (for full experimental details see supplementary methods).

### Tissue collection

Mice were returned to their home cage after the 30 min restraint stress and then killed by cervical dislocation after a further 60 min (90 min after onset of restraint stress). Brains were immediately removed and placed in 4% paraformaldehyde (PFA) solution prepared in phosphate buffer saline (PBS) overnight and then transferred to a 25% sucrose solution (Sigma-Aldrich, UK) prepared in PBS. They were then frozen in OCT (Cryomatrix, Thermofisher) and stored at −20°C.

### c-Fos immunohistochemistry

Brains were sliced in 40 µm coronal sections using a freezing microtome (Reichert, Austria). Sections were blocked for 30 mins in TBS-T (Trizma buffer saline with 0.1% Triton X) with 3% normal goat serum (Vector Laboratories). Sections were incubated with primary antibody rabbit anti c-Fos (1:4000, ABE457, Merck Millipore) overnight at 4°C. Sections were then incubated with secondary antibody goat anti-rabbit (1:500, A32731, Alexa Fluor 488, Invitrogen) for 3 h and then with 4′,6-diamidino-2-phenylindole (DAPI) for a further 3 min (for full experimental details see supplementary methods).

Images were taken on a Leica widefield microscope with DFC365 FX camera with LasX software. The paraventricular nucleus of the hypothalamus (PVN) was captured with a 10× objective and the central (CeA) and basolateral amygdala (BLA) with a 5× objective. A picture of the PVN was taken across three different sections, bregma level −0.58 to −0.94 mm. A picture of the amygdala (left or right) was taken across three different sections, bregma level (−1.22 to −1.40 mm). Some brains/sections were lost due to tissue damage. *N* = 10 per age group amygdala was obtained. *N* = 10 PVN for aged and young group were obtained, *N* = 9 for middle-aged group. c-Fos positive cells that co-localised with DAPI were counted manually using ImageJ cell counter. Contrast was adjusted uniformly across all images. c-Fos count was normalised by dividing c-Fos count by area of region.

### Data analysis

Where data were normally distributed (tested using the Shapiro–Wilk test) or required two-factor repeated measures (RM) analysis, RM two-way analysis of variance (ANOVA), one-way ANOVA, or independent samples *t*-test were performed where appropriate. Where normality was violated, the Kruskal-Wallis test or Mann–Whitney *U*-test were performed. Where significant main effects or interactions were observed (*p* < 0.05), these are reported in the results section with appropriate post hoc pairwise comparisons to explore age differences. Where main effect or interaction statistics found a trend-level effect (*p* < 0.1), these are mentioned in the text but were not further analysed. When conducting ANOVA tests, where data did not meet the assumption of sphericity, the Huynh–Feldt correction was used to adjust degrees of freedom. When conducting single independent *t*-tests, Levene’s Test for Equality of Variances was used to determine whether equal variances were assumed with correction being applied if this was not valid. Statistical outliers were defined as values 2 standard deviations away from the group mean. Analysis was performed on IBM SPSS statistics v. 24 for Windows and all graphs were made using GraphPad Prism 8.3.0 for Windows.

## Results

### Aged mice show reduced exploration of a novel arena but show no deficit in the novel object recognition task

**Figure 2.**
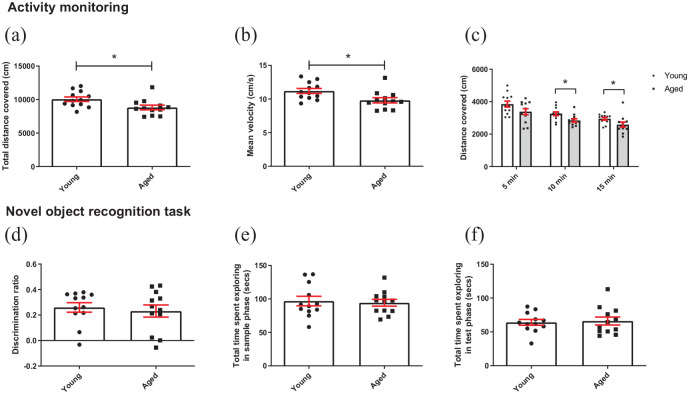
Aged mice show reduced exploration in a novel arena but normal cognition in the novel object recognition test: (a) aged mice covered less distance overall in a novel environment compared to younger mice (independent *t*-test), (b) aged mice were slower than younger mice (independent *t*-test), (c) distance covered was not different between age groups in the first 5 mins of the task but was by 10 and 15 mins. Mice also covered less distance over time (RM two-way ANOVA with pairwise comparisons). (d) There was no difference in discrimination ratio during the test phase of the NOR task. Bars are mean ± SEM, with data points overlaid. (e) There was no difference in time spent exploring objects in the sample phase of the NORT between age groups, (f) or in the test phase. *p < 0.05, ###*p* < 0.001—refers to main effect of time. *N* = 12 per age group.

In a novel arena, aged mice covered less distance and had a slower mean velocity than younger mice (*t*_(22)_ = 2.536, *p* = 0.019 and *t*_(22)_ = 2.613, *p* = 0.016, respectively, independent *t*-test) **(**Figure 2(a) and (b)**)**. When data were split into 5-min time bins, there was a main effect of time (*F*_(2,44)_ = 35.330, *p* = 7.06 × 10^−10^, RM two-way ANOVA) where distance travelled reduced with time. There was also a main effect of age group (*F*_(1,22)_ = 6.434, *p* = 0.019), but there was no age group*time interaction (*p* > 0.05). Pairwise comparisons revealed no difference in distance travelled between age groups in the first 5 mins, but a difference emerged in the second- and third-time bin *p* = 0.013 and *p* = 0.049, respectively **(**Figure 2(c) **)**.

In the NOR task, there was no difference in discrimination ratio between age groups (*p* > 0.05) (Figure 2(d)), and there was no difference in exploration of the objects between age groups during the sample phase (Figure 2(e)) or test phase (Figure 2(f)).

### Aged mice show changes in motivated behaviour

**Figure 3.**
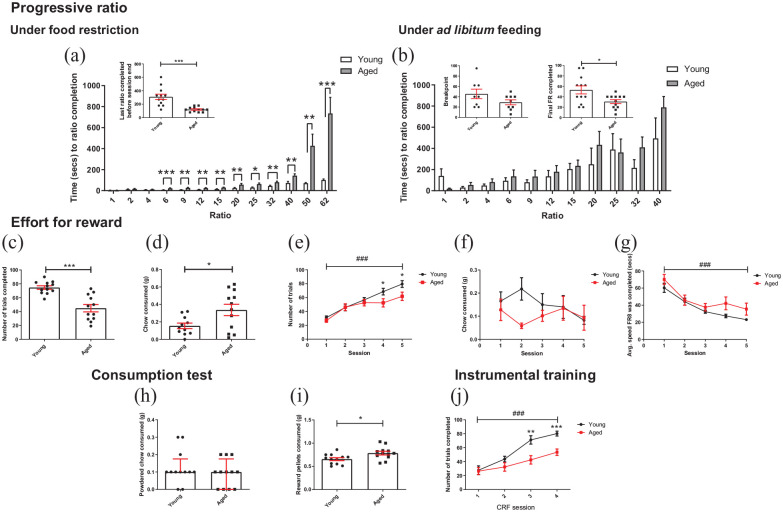
Aged mice show changes in motivation under different conditions: (a) under food restriction, aged mice completed the task on a lower final ratio in the progressive ratio schedule than younger mice (independent *t*-test). Younger mice completed ratios 6–62 faster than aged mice (RM two-way ANOVA with pairwise comparisons). (b) Under *ad libitum* feeding conditions and for animals which achieved a true breakpoint, there was no difference between age groups (independent *t*-test). However, when all mice were included in the analysis, aged mice finished on a lower ratio than younger mice (independent *t*-test). There was no difference in speed across age groups under free feeding conditions (independent *t*-tests). (c) On first exposure to the EfR task, aged mice completed less trials than younger mice (independent *t*-test). (d) In the same session, aged mice consumed more chow (independent *t* test). (e) When the task was repeated over five consecutive days and at the end of the PR training and testing, aged mice completed less trials than younger mice only in sessions 4 and 5. Number of trials completed increased across the week in both age groups (*p* < 0.05, *p* < 0.001, RM two-way ANOVA with pairwise comparison). (f) There was no effect of session or age group on chow consumption. (g) Mice became faster at completing trials over sessions (*p* < 0.001, RM two-way ANOVA). (h) In a 10 min consumption test, there was no difference in chow consumed between age groups (Mann–Whitney *U* test, bars are median ± interquartile range). (i) Aged mice consumed more reward pellets than younger mice (independent *t*-test). (j) Aged mice completed less trials in CRF training session 4 and 5 (RM two-way ANOVA with pairwise comparison). Bars are mean ± SEM with data points overlaid (unless otherwise indicated). *p < 0.05, **p < 0.01, ***p < 0.001, ###*p* < 0.001—refers to main effect of session. *N* = 10–12 per group.

Under food restriction, aged mice ended the PR session at a lower final ratio completed (*t*_(12.464)_ = 4.563, *p* = 0.001, independent *t*-test); however, mice failed to reach a true breakpoint, that is, they kept working until session time limit. Analysis of time taken to complete each ratio within the PR session showed a main effect of ratio (*F*_(1.403, 30.865)_ = 22.812, *p* < 0.0001, RM two-way ANOVA), where time taken to complete a ratio increased with ratio. There was also a ratio*age group interaction (*F*_(1.403, 30.865)_ = 13.581, *p* < 0.0001) and a main effect of age (*F*_(1,22)_ = 19.960, *p* < 0.0001). Ratio 62 was the final ratio all mice completed so was used as a cut off for analysis. Pairwise comparisons showed younger mice completed ratio 6–62 faster than aged mice (*p* ⩽ 0.016) (Figure 3(a)). Under *ad libitum* feeding conditions eight young mice and nine aged mice reached breakpoint, that is, ended session with 5 mins of inactivity. There was no difference in breakpoint between age groups (*p* > 0.05). However, when all mice were added to the analysis, including those that did not reach breakpoint, a difference emerged (*t*_(16.108)_ = 2.619, *p* = 0.019). There was no difference in time taken to complete each ratio between age groups (up to ratio 40). RM ANOVA was not possible for this analysis as there were missing data points due to animals completing different final ratios before the break point or end of the session. As the main interest was the effect of age group, a series of independent *t*-tests were conducted comparing the speed to ratio completion for each ratio independently and for old versus young mice. There was no difference in speed to ratio completion between age groups (*t*_(15)_ ⩽ 1.565, *p* ⩾ 0.113, independent *t*-tests) (Figure 3(b)).

On first exposure to the EfR task, aged mice obtained less of the high value reward pellets than younger mice (*t*_(15.982)_ = 5.088, *p* = 0.0001, independent *t*-test) but consumed more of the low value chow (*t*_(15.819)_ = 2.453, *p* = 0.023). One younger mouse was excluded as an outlier (Figure 3(c) and (d)). In a subsequent 5-day test, analysis of number of trials completed over sessions showed a main effect of session (*F*_(3.22, 70.833)_ = 41.553, *p* < 0.0001, RM two-way ANOVA), and there was a session*age group interaction (*F*_(3.22, 70.833)_ = 2.73, *p* = 0.047). There was a trend-level effect of age (*F*_(1,22)_ = 3.038, *p* = 0.095). Post hoc pairwise comparison showed younger mice completed more trials only in the final two sessions (*p* = 0.046 and *p* = 0.032, respectively) (Figure 3(e)). However, there was no effect of session or group on consumption of chow (*p* > 0.05) although there was a trend towards a chow*age group interaction (*F*_(3.59, 75.4)_ = 2.237, *p* = 0.08) (Figure 3(f)). *n* = 1 young mouse was excluded for digging in chow over consecutive sessions. In a single instance of digging, value was replaced with group mean to permit RM ANOVA analysis (*n* = 1 young mouse). Analysis of average speed (speed at which FR8 was completed, averaged over a session) across the sessions showed a main effect of session on speed, where speed increased over sessions in both age groups (*F*_(2.302, 50.636)_ = 34.476, *p* < 0.0001). However, there was no age*session interaction or effect of age (*p* > 0.05) (Figure 3(g)). Consumption tests showed aged mice consumed more reward pellets in 10 mins than younger mice (*t*_(22)_ = 2.587, *p* = 0.0168, independent *t*-test) while consumption of chow did not differ (*p* > 0.05) (Figure 3(h) and (i)).

Analysis of CRF training performance revealed a main effect of session on number of trials completed (*F*_(3,66)_ = 44.471, *p* < 0.0001, RM two-way ANOVA), as well as a session*age group interaction (*F*_(3,66)_ = 5.815, *p* = 0.0014) and a main effect of age (*F*_(1,22)_ = 8.365, *p* = 0.008). There was no difference in performance between groups in the first two sessions, but younger mice completed more trials in the final two sessions (*p* ⩽ 0.003) (Figure 3(j)). There was no difference in session at which FR training was completed between age groups (Mann–Whitney *U*-test, *U* = 54.50, *p* = 0.3058) (Supplementary data S1).

### Aged mice show changes in hedonic behaviour, anxiety-like behaviour and stress reactivity

**Figure 4.**
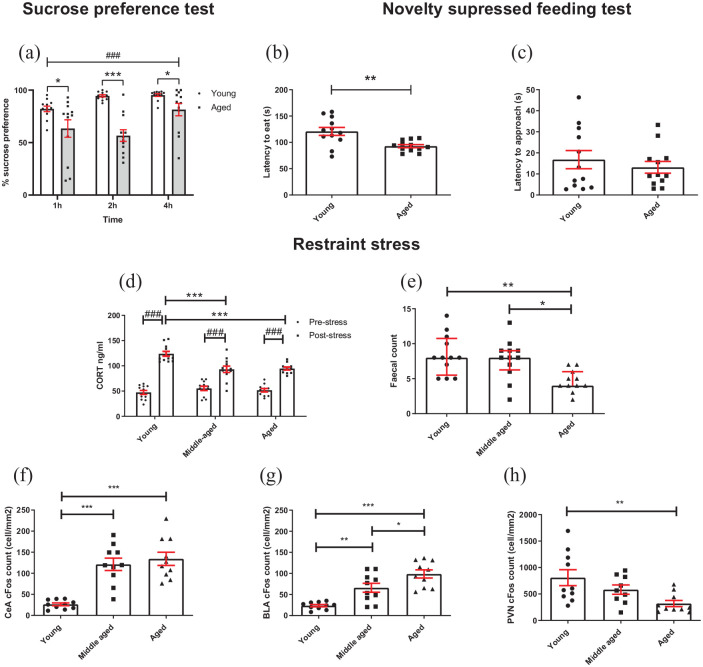
Aged mice show changes in reward sensitivity and stress reactivity: (a) aged mice showed a reduced sucrose preference compared to younger mice across all time points, and preference changed across time (RM two-way ANOVA with pairwise comparisons). (b) In the NSFT, aged mice took less time to eat from the bowl than younger mice (independent *t*-test). (c) There was no difference in latency to approach bowl between age groups (independent *t*-test). (d) Following 30-min restraint stress, CORT was increased in all groups, but aged mice showed a reduced CORT response to stress compared to younger mice but not middle aged (RM two-way ANOVA with pairwise comparisons). (e) Aged mice had a lower faecal count following stress compared to young and middle-aged groups (Kruskal–Wallis test with Dunn’s post hoc (bars are median with interquartile range)). (f) Following stress, middle-aged and aged mice had a greater c-Fos count in the CeA compared to younger mice (*p* < 0.001, one-way ANOVA with Tukey’s post hoc analysis). (g) Aged mice had a greater BLA c-Fos count compared to middle-aged and young mice. Younger mice had a lower c-Fos count in the BLA compared to middle-aged mice (one-way ANOVA with Tukey’s post hoc analysis). (h) Younger mice had a greater c-Fos count in the PVN compared to aged mice (one-way ANOVA with Tukey’s post hoc analysis). Unless otherwise indicated bars are mean ± SEM with data points overlaid. *p < 0.05, **p < 0.01, ***p < 0.001, ###*p* < 0.001—refers to main effect of time or pairwise effect of stress. *N* = 9–12 per group.

Analysis of % sucrose preference showed a main effect of time (*F*_(1.781, 39.191)_ = 12.146, *p* < 0.0001, RM two-way ANOVA) where sucrose preference increased with time. There was also a time*age group interaction (*F*_(1.781, 39.191)_ = 7.076, *p* = 0.003) and an effect of age group (*F*_(1,22)_ = 16.692, *p* < 0.0001) (Figure 4(a)). There were three leaks in water sacks during testing (2 aged, 1 young) and where this occurred the value for this sack was replaced with the group mean to permit RM ANOVA analysis. Post hoc pairwise comparison showed younger mice had a higher sucrose preference than aged mice at each time point (*p* ⩽ 0.043). There was a main effect of time on total liquid consumed in the SPT (two-way RM ANOVA, *F*_(2,44)_ = 70.192, *p* < 0.0001, a time*age group interaction (*F*_(2,44)_ = 14.672, *p* < 0.0001 as well as a main effect of age group (*F*_(1,22)_ = 26.977, *p* < 0.0001). Pairwise comparison revealed that young and aged mice drank an equivalent volume of liquid in the first hour of the test (*p* > 0.05) but aged mice drank less liquid at hour 2 and 4 of the test (*p* < 0.0001) (Supplementary data S2A). These differences in total consumption were accounted for in the % sucrose calculation. During habituation to the SPT, there was a main effect of habituation day on total liquid consumed (two-way RM ANOVA, *F*_(2,44)_ = 29.950, *p* < 0.0001). There was also a habituation day*age group interaction (*F*_(2,44)_ = 4.44, *p* = 0.018) and a main effect of age group (*F*_(1,22)_ = 6.032, *p* = 0.022). Pairwise comparison revealed that aged and young mice drank equivalent volumes on Days 1 and 2 of habituation (*p* > 0.05) but young mice drank more liquid on the final day of habituation (*p* < 0.0001) (Supplementary data S2B). Aged mice took less time to eat from the bowl than younger mice in the NSFT (*t*_(4.208)_ = 3.411, *p* = 0.004, independent *t*-test) (Figure 4(b)) but latency to approach the bowl was not different (*p* > 0.05) (Figure 4(c)). Mice were weighed before and directly after the 24-h food restriction. Younger mice lost a greater percentage of their body weight compared to aged mice (unpaired *t*-test, *t*(22) = 5.654, *p* < 0.0001; Supplementary data S3).

Under acute restraint stress, baseline corticosterone was similar for all age groups but aged animals had a lower corticosterone response to restraint (main effect of stress *F*_(1,32)_ = 179.608, *p* < 0.0001, age F_(1,32)_ = 5.772, *p* = 0.007, and stress*age group interaction *F*_(2,32)_ = 9.862, *p* = 0.0005, RM two-way ANOVA). Pairwise comparison revealed CORT was increased following stress in all age groups, but this was higher in the young mice (young vs aged or middle aged: *p* = 0.001 and *p* = 0.0003, respectively) (Figure 4(d)). There was also an effect of age on faecal count (*H*_(2)_ = 12.424, *p* = 0.002, Kruskal–Wallis test). Dunn’s post hoc test showed aged mice had a lower faecal count than middle-aged mice (*p* = 0.011) and young mice (*p* = 0.004) (Figure 4(e)). Post-mortem analysis of neuronal activation following restraint stress showed CeA c-Fos count was higher in both the aged and middle-aged animal (main effect of age *F*_(2,29)_ = 22.102, *p* < 0.0001, one-way ANOVA) with Tukey’s post hoc analysis (*p* < 0.0001 vs younger mice) (Figure 4(f)). There was also an effect of age on BLA c-Fos count (*F*_(2,29)_ = 19.915, *p* < 0.0001), where middle-aged and young mice had a lower BLA c-Fos count than aged mice (*p* = 0.029 and *p* < 0.0001, respectively). Middle-aged mice also had a higher BLA c-Fos count than younger mice (*p* = 0.004) (Figure 4(g)). PVN c-Fos count was higher in younger mice compared to aged mice (main effect of age *F*_(2,28)_ = 5.326, *p* = 0.012, Tukey’s post hoc, *p* = 0.008) (Figure 4(h)).

## Discussion

Aged male mice displayed behavioural deficits in both motivation and affect which suggests they may develop a phenotype with deficits in behavioural domains relevant to apathy. Using measures related to motivation, we observed that aged mice showed a more rapid decline in activity in a novel environment, responded with reduced vigour in the PR task and were more likely to choose a low-value low-effort reward under certain conditions. These effects did not appear to be related to changes in appetite, cognition or gross changes in locomotor function. When we reduced motivation across all age groups using free feeding, we found that the deficit in the aged animal’s performance in the PR task related mainly to rate of responding. In the NSFT, aged mice exhibited lower levels of anxiety-related behaviour and were faster to consume the food; however, they also showed reduced reward sensitivity contrasting with effects typically observed in rodent models of depression (Planchez et al., 2019). When we consider these changes in emotional behaviour alongside the blunted corticosterone and PVN c-Fos activation in response to restraint stress, we propose that aged mice show emotional blunting. The following discussion considers these two domains associated with the psychiatric symptom of apathy and the potential to use aged mice to better understand the underlying neurobiology.

### Aged mice show a reduction in motivated behaviours

Aged mice were slower and covered less distance in the open-field arena. When the analysis was broken down by time bin, the effect was more apparent after 10 and 15 min, suggesting that initial exploration of the environment and locomotor function did not differ. However, it should be noted the effects were marginal. These effects may be due to declining motivational drive to explore, though it is important to acknowledge that age-related deterioration in motoric capability and fatigue may also play a role. Ageing has been associated with psychomotor slowing (Tombaugh, 2004), where slowing is due to a reduction in motivation and emotional arousal, which can be independent of age-related effects (Seidler et al., 2010).

During operant conditioning aged animals initially responded at a similar level but then failed to increase their rate of responding to the same level as the younger animals over time. However, both age groups showed learning across sessions, suggesting reduced vigour rather than an overt learning impairment. In the initial studies carried out under food restriction, aged animals appeared less motivated and completed less ratios for reward under a PR schedule of reinforcement. They were slower to complete each ratio but a true breakpoint, in which the mouse gives up before the session ended was not reached. Some PR studies are limited by time rather than lack of responding (Heath et al., 2015) or a combination of both (Gourley et al., 2016). This may mean that conclusions are confounded by changes in rate of response and an assumption that the last ratio completed before the task times out is also break point. When the motivational state was reduced by providing food *ad libitum*, we found no difference in breakpoint or the speed of ratio completion. This suggests rate of responding is driving the motivational deficit in aged mice and the effects are more apparent when control animals are in a high motivational state. These findings are in line with studies using a Huntington’s disease (HD) model, in which apathy is a core symptom, where they found a reduction in rate of response in a PR schedule of reinforcement, relative to controls (Heath et al., 2019; Oakeshott et al., 2012). This was consistent with a study using HD patients with apathy, and apathy questionnaire scores negatively correlated with breakpoint scores (Heath et al., 2019).

An alternative way to look at motivation is the EfR task, an effort-based decision-making (EBDM) task. By offering a choice to the mouse, this task provides a more complex measure of motivation involving economic choices such as cost/benefit analyses (Salamone et al., 2018). In this task, a shift from the high-effort to low-effort reward option is thought to indicate a change in motivation whereas an overall suppression of consumption would indicate a change in appetite. The integration of the findings across both PR and EfR has been used to try to address limitations associated with any one task in isolation. In an initial single test session following FR training, aged mice completed less trials but consumed more chow, indicating preference for the lower effort option compared to younger mice. However, after animals had undergone multiple sessions under FR8 and PR schedules and, when the task was presented over consecutive days, an age difference emerged only in the final two sessions. There is a potential confound with this format which links to our PR data. Animals were run under food restriction, but mice are given food *ad libitum* over the weekend and food restricted across the week. In this second study, changes in chow consumption were not observed; however, variability was higher, possibly resulting from excessive digging/waste from the chow bowl. Changes in EBDM have been reported in Schizophrenia patients and Parkinson’s disease patients with apathy (Chong et al., 2015; Le Heron et al., 2018). However, much of pre-clinical work on this type of EBDM focusses on investigating the role of the dopaminergic system, rather than probing for apathy behaviour in disease/ageing models (Le Heron et al., 2019).

Together, these data suggest aged male mice have a deficit in the activational phase of motivated behaviour, which is characterised by speed, vigour and persistence (Salamone et al., 2016). These changes appear not to be explained by changes in appetite or a cognitive impairment as no differences were observed in consumption tests or in the NOR task. There is conflicting evidence in the human literature whether motoric vigour is reduced in apathy, where some studies suggest vigour in pursuit of reward may be conserved in apathy patients, while other work points to a reduction (Le Heron et al., 2019).

### Aged mice show emotional blunting

Rodents are known to supress feeding in response to novelty and the time taken to eat in this novel context provides a behavioural readout of this stress response. The NSFT is based on this principle and has been used for decades in the pre-clinical testing of anxiolytics and antidepressants (Nestler and Hyman, 2010; Shephard and Broadhurst, 1982) and in phenotypic studies of depression models including ELA and chronic social defeat stress (Stuart et al., 2019; Venzala et al., 2012). In the NSF test, aged mice were quicker to consume food in a novel environment, suggesting lower novelty-induced hyponeophagia. These findings are consistent with reduced anxiety and contrast effects seen in rodent models of depression where increased feeding latencies are consistently observed (Planchez et al., 2019). While young and aged mice ate equivalent levels of chow in a separate consumption test, younger mice lost a greater proportion of their body weight following food restriction. Therefore, there is a potential for metabolic differences that may influence appetite and therefore impact measures of motivation driven by hunger. The quantification of ghrelin/leptin levels could provide further information on potential differences in appetite. Conversely, aged animals had reduced reward sensitivity in the SPT, similar to the effects observed in rodent models of depression (Willner, 2016). Human studies have shown reward sensitivity deficits in ageing (Muhammed et al., 2016). A probabilistic reinforcement learning task showed monetary loss had a larger impact on subsequent behaviour than gain in elderly versus younger participants (Hämmerer et al., 2010). This disruption may be due to age-related changes to the efficiency of dopaminergic and serotonergic neuromodulation (Eppinger et al., 2011; Schott et al., 2007). These behavioural findings provide an interesting contrast to depression-like phenotypes and importantly suggest emotional blunting rather than negative affective state.

Studies in the ELA model of depression have shown that stress reactivity is linked to deficits in the NSFT (Stuart et al., 2019; Uchida et al., 2010). To explore the apparent reduction in stress reactivity seen in the NSF test at a physiological level, we used an acute restraint stress which reliably increases the stress hormone corticosterone (CORT) (Harizi et al., 2007; Nohara et al., 2016). There were no differences in CORT under baseline conditions and all age groups showed an increase following 30-min restraint. However, in line with behavioural findings, we show aged mice had a blunted stress-induced CORT response and the oldest group also had a reduced faecal count consistent with reduced stress reactivity. We also found age-related changes in the response to stress centrally, where c-Fos activation in the paraventricular nucleus of the hypothalamus (PVN) was reduced in the 24 mo group compared to the youngest group, yet central (CeA) and basolateral amygdala (BLA) activation was increased in the aged groups. The PVN is a core part of the stress response, its activation results in the release of CORT (Benarroch, 2005). This reduced activation could explain the reduced CORT response in the aged mice. The CeA plays a key role in the stress response, integrating behaviour and autonomic response to aversive stimuli (Day et al., 2005). Previous research has shown that there is relatively little activation of this region in response to processive stressors and is more responsive to homeostatic disruption or systemic stress (Day et al., 2005). However, aged mice show greater activation of the BLA/CeA during stress compared to the young mice. The reason for this and its functional significance is unclear. However changes to the functional gating of limbic information by local PVN projections may explain age-related changes to the HPA axis (Herman et al., 2002). Stress reactivity and active coping in response to aversive experiences has previously been shown to reduce with age in mice (Oh et al., 2018) as well as rat and human studies (Brugnera et al., 2017; Buechel et al., 2014). There is some variability in findings possibly due to a lack of standardisation across studies including the nature and intensity of the stressor (Novais et al., 2017; Segar et al., 2009). An interesting next step would be to test whether this blunted physiological response to an acutely aversive event translates to changes in aversive learning in a contextual fear conditioning paradigm.

It is important to acknowledge that only male mice were used in this study and translatability of findings would benefit from the use of female mice to account for any potential sex differences in the behavioural domains tested. While sex differences in motivated behaviour have been reported (Song et al., 2018), it is unclear if there is a sex difference in how ageing affects these behaviours, with the exception of exploratory behaviour where parallel declines have been observed (Adelöf et al., 2019).

## Conclusion

It is not possible to fully translate the psychiatric symptom of apathy to non-human animals, and there is no one behavioural test that will tell us whether an animal is apathetic. However, by using a battery of behavioural tests that probe aspects of motivated and emotional behaviour we show that aged male mice have deficits in behaviours relevant to multiple domains of apathy. Specifically, deficits measured by the exploration of a novel arena, the PR task and the EfR task indicate a reduction in motivated behaviour. A reduction in stress reactivity and reduced reward sensitivity provide evidence for emotional blunting in aged mice. Together, these data suggest naturally aged mice have the potential to provide a model to investigate the underlying neurobiology of behaviours relevant to apathy. The findings relating to stress reactivity add to previously published work (Oh et al., 2018) and raises an important issue when considering data obtained from stress-driven cognitive tasks such as the commonly used Morris Water Maze (Morris, 1981). Changes in stress reactivity could influence learning independent of a specific cognitive impairment.

## Supplemental Material

sj-docx-1-bna-10.1177_23982128211015110 – Supplemental material for Evidence for deficits in behavioural and physiological responses in aged mice relevant to the psychiatric symptom of apathy Click here for additional data file. Supplemental material, sj-docx-1-bna-10.1177_23982128211015110 for Evidence for deficits in behavioural and physiological responses in aged mice relevant to the psychiatric symptom of apathy by Megan G. Jackson, Stafford L. Lightman, Gary Gilmour, Hugh Marston and Emma S. J. Robinson in Brain and Neuroscience Advances

sj-docx-2-bna-10.1177_23982128211015110 – Supplemental material for Evidence for deficits in behavioural and physiological responses in aged mice relevant to the psychiatric symptom of apathy Click here for additional data file. Supplemental material, sj-docx-2-bna-10.1177_23982128211015110 for Evidence for deficits in behavioural and physiological responses in aged mice relevant to the psychiatric symptom of apathy by Megan G. Jackson, Stafford L. Lightman, Gary Gilmour, Hugh Marston and Emma S. J. Robinson in Brain and Neuroscience Advances
