# Supplementary material for: Evidence for deficits in behavioural and physiological responses in aged mice relevant to the psychiatric symptom of apathy
Source: Brain Neurosci Adv. 2021 May 25;5:23982128211015110. doi: 10.1177/23982128211015110 (PMC8161852; doi:10.1177/23982128211015110)
Supplement: sj-docx-1-bna-10.1177_23982128211015110 – Supplemental material for Evidence for deficits in behavioural and physiological responses in aged mice relevant to the psychiatric symptom of apathy [file sj-docx-1-bna-10.1177_23982128211015110.docx]

Supplementary data

***S1 Session at which fixed ratio training was completed.*** *There was no significant difference in time taken to complete fixed ratio training between young and aged mice (Mann-Whitney U Test, p > 0.05). Bars are median ± interquartile range with individual data points overlaid. N = 12 per group.*


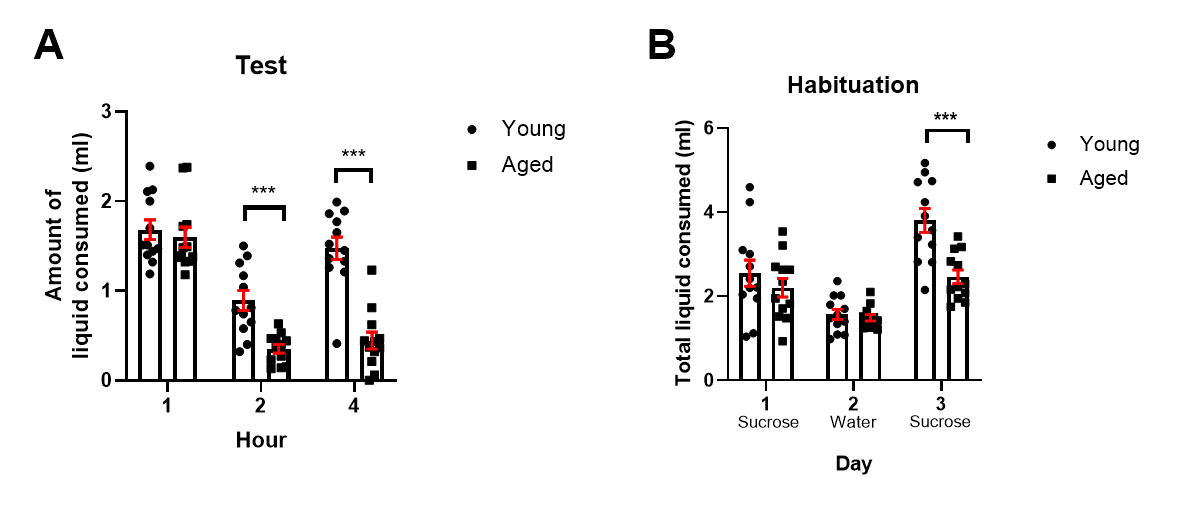


***S2 Liquid volume consumed during habituation and test days. A*** *Young and aged mice drank equivalent volumes in the first hour of the task, but young mice drank more at hour 2 and 4 of the test (RM two-way ANOVA with pairwise comparison, p < 0.0001).*  ***B*** *Young and aged mice drank equivalent volumes of liquid in the first two days of habituation but young mice drank more on the third day (RM two-way ANOVA with pairwise comparison, p < 0.0001). Bars are mean ± SEM with individual data points overlaid. N = 12 per group.*

***S3 % body weight loss following 24-hour food restriction.*** *Younger mice lost a greater percentage of their body weight compared to aged mice following a 24-hour food restriction (independent t-test, p < 0.0001). Bars are mean ± SEM with individual data points overlaid. N = 12 per group.*
